# Drastic transitions of excited state and coupling regime in all-inorganic perovskite microcavities characterized by exciton/plasmon hybrid natures

**DOI:** 10.1038/s41377-021-00701-8

**Published:** 2022-01-02

**Authors:** Shuki Enomoto, Tomoya Tagami, Yusuke Ueda, Yuta Moriyama, Kentaro Fujiwara, Shun Takahashi, Kenichi Yamashita

**Affiliations:** Faculty of Electrical Engineering and Electronics, Kyoto Institute of Technology, Matsugasaki, Sakyo-ku, Kyoto, 606-8585 Japan

**Keywords:** Polaritons, Microresonators, Photonic devices, Polaritons

## Abstract

Lead-halide perovskites are highly promising for various optoelectronic applications, including laser devices. However, fundamental photophysics explaining the coherent-light emission from this material system is so intricate and often the subject of debate. Here, we systematically investigate photoluminescence properties of all-inorganic perovskite microcavity at room temperature and discuss the excited state and the light–matter coupling regime depending on excitation density. Angle-resolved photoluminescence clearly exhibits that the microcavity system shows a transition from weak coupling regime to strong coupling regime, revealing the increase in correlated electron–hole pairs. With pumping fluence above the threshold, the photoluminescence signal shows a lasing behavior with bosonic condensation characteristics, accompanied by long-range phase coherence. The excitation density required for the lasing behavior, however, is found to exceed the Mott density, excluding the exciton as the excited state. These results demonstrate that the polaritonic Bardeen–Cooper–Schrieffer state originates the strong coupling formation and the lasing behavior.

## Abstract (web summary)

All inorganic lead-halide perovskite microcavity forms a light–matter hybrid state based on long-range correlated e–h pairs.

## Graphical abstract

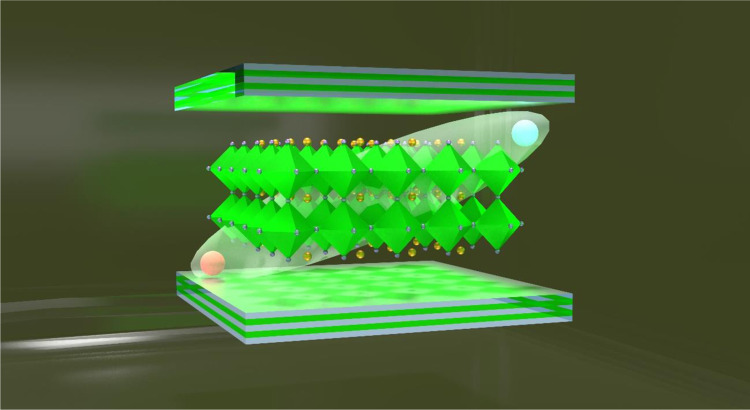

## Introduction

Recent advances in research fields on lead-halide perovskites have revealed great potential of this material system for applications in various optoelectronic technologies, such as photoelectric conversion and electroluminescence^1,2^. Excellent optoelectronic properties of the perovskites, such as the wide variation in bandgap^3,4^ and high photoluminescence quantum yield^5,6^, offer a considerable prospect as lasing devices with various functionalities^7,8^, e.g., color tunability and low-power consumption. Coherent-light emissions from many types of resonators including microcavity^5,6^, distributed feedback^9,10^, and whispering gallery modes (WGM)^11–13^ have been demonstrated with perovskite materials in the forms of thin films^5,7–10^, microcrystals^10–12^, and colloidal nanocrystals^13^. In addition to conventional “photon lasing”, some studies have suggested “polariton lasing” in the perovskite microcavities^14,15^, where an optical cavity mode and an exciton transition dipole moment are strongly coupled to form a polariton quasi-particle^16–20^. In particular, two-dimensional (2D) layered perovskites and Cl- and Br-based three-dimensional (3D) perovskites show exciton binding energies above the activation energy of room temperature (RT), and thus these materials are expected to condense the polariton particles at room temperature. The macroscopically coherent state of the condensed polariton^19–21^ is a promising platform for quantum information science^22^, e.g., quantum simulation^23^, quantum-information processing^24^, and single-photon emission^25^.

**Fig. 1:**
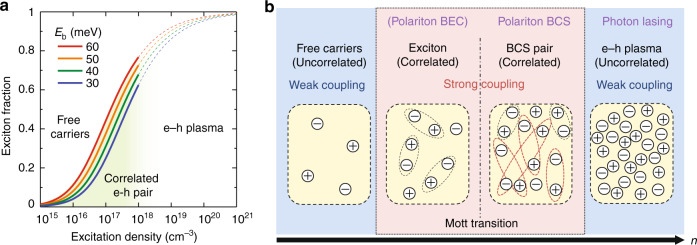
Excited states expected for CsPbBr_3_ microcavity at RT. **a** Exciton fraction *x/n* as a function of total excitation density *n* calculated from Saha relationship (Eq. (S1.1) in Supplementary Information). **b** A schematic revealing transitions of populated excitation particles and coupling regimes varied with increased excitation densities

Despite a great promise as a platform for RT polaritonic devices, the underlying physics of lasing phenomena in 3D perovskite are still controversial. In particular, there are various claims about excited species that contribute to the lasing in CsPbBr_3_ crystals^8,26–28^. Zhu et al. reported that CsPbBr_3_ crystalline nanowires exhibit a Fabry-Pérot type normal photon lasing due to stimulated emission of electron–hole (e–h) plasmas^28,29^. On the other hand, Xiong et al. clearly revealed light–matter strong coupling in CsPbCl_3_ and CsPbBr_3_ microcavities and claimed polaritonic lasing based on Bose–Einstein condensation (BEC)^14,30^. These conflicting claims raise a question of what is the excited species that induces the lasing emission; uncorrelated e–h pairs, excitons, or something else. The exciton binding energy of CsPbBr_3_ (30–60 meV) is comparable to the activation energy of RT^28^. For such a case, as shown in Fig. 1a and Note 1 in Supplementary Information, Saha equation expects that correlated and uncorrelated e–h pairs, i.e., exciton and free carriers, respectively, coexist in thermal equilibrium at the excitation density within a range where the lasing occurs (10^17^–10^18 ^cm^−3^)^26,31^. To further complicate matters, this excitation density range may even lead to the Mott transition^32^, which denotes a phase transition from insulating excitonic phase to metallic e–h plasma phase.

As an intermediate regime between the exciton BEC and the photon lasing due to the e–h plasma, Bardeen–Cooper–Schrieffer (BCS) state has been proposed (Fig. 1b)^33,34^. In this intermediate regime, the excitation density is high enough to screen the Coulomb interaction of strongly bound excitons, but low enough to allow Cooper-pair-like e–h pairs. Above a certain excitation density, the coherent length of the Cooper-pair-like e–h pairs exceeds the interparticle separation, and then a BCS-type condensation is expected to occur^33^. Theoretical studies have revealed that the BEC–BCS crossover is a smooth transition despite their quite different physical origins^33,35^. Furthermore, it is reported in inorganic semiconductor microcavities that the strong light field can strengthen the Cooper-pair-like e–h pairing (i.e., polaritonic BCS)^36–38^, leading to maintaining the BCS regime even at high excitation density^39,40^. This polariton BCS scenario might be possible to explain the recent experimental results of CsPbBr_3_ microcavities comprehensively, but there is no systematic study from this physical aspect.

Here, we systematically investigate excited states in CsPbBr_3_ microcavity using a set of photoluminescence (PL) measurements under both below- and above-threshold conditions. By comparing the results of a bare crystal microplate with a microcavity, we show that the microcavity undergoes the polariton BCS regime. A central finding of this study is the observation of a clear transition from weakly coupling regime to strong coupling regime, which can be seen as a change in the PL dispersion curve depending on below-threshold pumping fluence. This result demonstrates a change in the dominant excited species from uncorrelated e–h pair to exciton. At the pumping fluence above the threshold, the CsPbBr_3_ microcavity shows a laser-like emission with bosonic energy condensation characteristics. On the other hand, a cavityless CsPbBr_3_ crystalline microplate under this fluence range exhibits PL features attributed to the Mott transition, indicating a polaritonic BCS state as the origin of the lasing phenomenon in the CsPbBr_3_ microcavity.

## Results

**Fig. 2:**
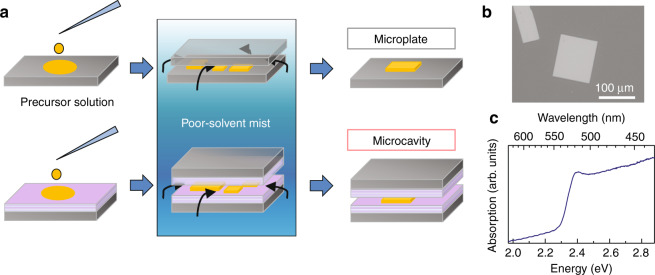
Space-limited antisolvent vapor-assisted crystallization method for CsPbBr_3_ single-crystal microplate, and microcavity fabrications. **a** Schematics for preparing microplate (upper row) and microcavity (lower row) with the antisolvent vapor-assisted method. Perovskite precursor solution is prepared by solving CsBr and PbBr_2_ powders into DMSO. ACN is used as poor solvent mist. **b** A scanning electron microscopic image of CsPbBr_3_ microplates. **c** Absorption spectrum of bare microplate near the band edge

We obtain CsPbBr_3_ crystalline microplates by the antisolvent vapor-assisted crystallization method. Details of the growth procedure are described in “Materials and methods”. In short, as shown in the upper row of Fig. 2a, small amount of perovskite precursor solution (dimethyl sulfoxide, DMSO) is casted onto a silica substrate, and another silica plate covers the casted solution to limit the vertical space for crystal growth. The sample is put in poor solvent mist (acetonitrile, ACN) at RT. One day later, plate-like crystals with lateral size of ~100 μm grow (Fig. 2b). When we use a pair of distributed Bragg reflectors (DBRs) instead of the silica substrates, the sample forms a microcavity structure with the conventional configuration of vertical-cavity surface-emitting laser (VCSEL). We can control the thickness of microplate by limiting the vertical space with a pair of glass plates (i.e., space-limited antisolvent vapor-assisted crystallization method)^41,42^. Surface profile measurements show that the height of microplates is in a range of ~0.2–1.5 μm (Figs. S2a and S2b in Supplementary Information). Clear (100) facet appears on the crystal surface spontaneously. X-ray diffraction analysis shows the [100]-oriented growth of CsPbBr_3_ crystal with the cubic or orthorhombic phase (see section 1 and Fig. S2c in Supplementary Information)^43^. Figure 2c exhibits a resonant enhancement of an absorption peak at ~2.38 eV.

**Fig. 3:**
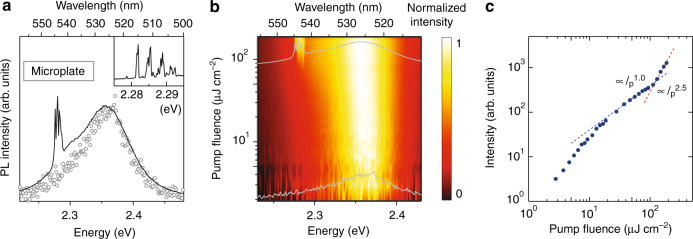
PL characteristics of CsPbBr_3_ bare microplate. **a** Normalized PL spectra under *I*_p_ of ~3.8 μJ cm^−2^ (open circle) and ~190 μJ cm^−2^ (solid line). The data are obtained by integrating the emission within azimuth of <~20°. Inset shows a high-resolution PL spectrum obtained at *I*_p_ of ~212 μJ cm^−2^. **b** 2D pseudo-color plot of normalized PL spectra as a function of pump fluence. As a help to reader, PL spectra obtained under *I*_p_ of 3.8 μJ cm^−2^ and 190 μJ cm^−2^ are also shown (gray curves). **c** Fluence-dependent emission intensity for one of the lasing modes (~2.276 eV). Blue and red dashed lines exhibit linear and super-linear functions representing below- and above-threshold responses, respectively

First, we show PL properties and lasing of a bare CsPbBr_3_ microplate. Time-integrated PL spectra under pulse pumping are shown in Fig. 3a. Note that these are results recorded using a ×10 objective with a numerical aperture of 0.3, in which the emission within the azimuth of < ~20° is integrated. At the pump fluence *I*_p_ of ~3.8 μJ cm^−2^, a PL signal with asymmetric spectral profile is observed in an energy range of ~2.3–2.4 eV. This is a typical spectral response of CsPbBr_3_ crystal at RT^31^, whereas the origin of asymmetric spectral shape is still under discussion^8,31,44,45^. Figure 3b exhibits a colormap of the *I*_p_-dependent normalized PL intensities. With increasing *I*_p_ (>~20 μJ cm^−2^), the PL signal shows a slight spectral change; i.e., growth of the shoulder on the low-energy side of the asymmetric spectral profile (~2.3 eV). Above *I*_p_ of ~90 μJ cm^−2^, the microplate sample exhibits a multimode-lasing that is similar to whispering gallery mode (WGM) lasing observed in previous studies^11,12^. However, the free spectral range of the observed modes (~28 cm^−1^) corresponds to the cavity length of ~150 µm (see the inset of Fig. 3a). This cavity length seems to be too large for a WGM mode of a 30 µm^2^ crystal shown in Fig. S3b in Supplementary Information. Therefore, random lasing is the most possible origin for the observed emission rather than the WGM lasing^46^. Figure 3c shows a threshold behavior in the fluence-dependent PL intensity detected at the lasing wavelength. These observations are very similar to previous studies, e.g., the work of Zhu et al., in which they assign this low-energy emission to the plasmonic emission and claim a normal photon lasing due to population inversion^44^. For our case, the density of the excited particle is estimated to be 1.6 × 10^18 ^cm^−3^ at *I*_p_ of ~10 μJ cm^−2^ (see Note 2 in Supplementary Information). Given that the Mott density was reported to be in a range of 1.8–4.7 × 10^17 ^cm^−3^^28,29,32,45,47,48^, our results also support the e–h plasma lasing for the bare CsPbBr_3_ microplate.

**Fig. 4:**
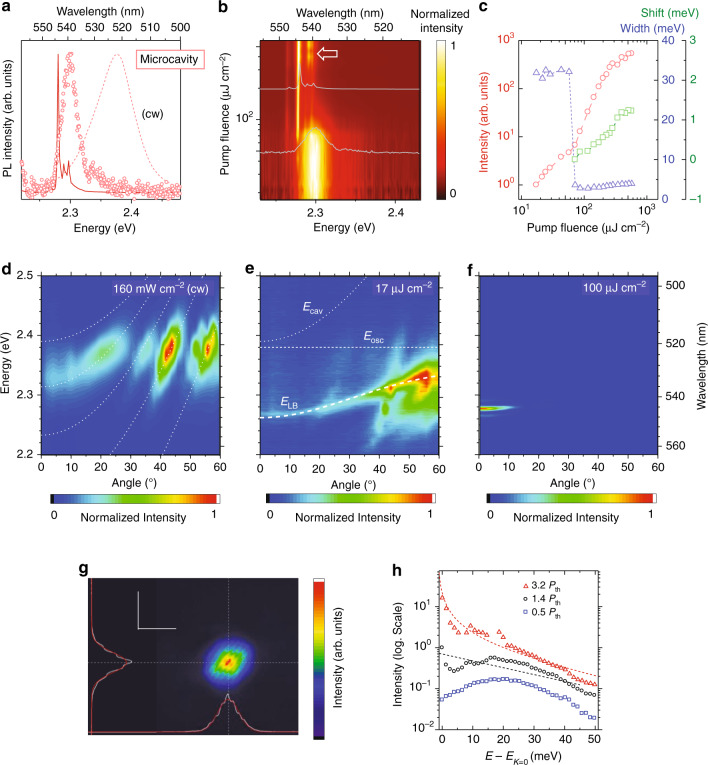
PL characteristics of CsPbBr_3_ microcavity. **a** Normalized PL spectra under *I*_p_ of ~17 μJ cm^−2^ (open circle) and ~200 μJ cm^−2^ (solid curve). The data are obtained by integrating the emission of radiation angles within < ~20°. Dashed curve shows a result under cw excitation. **b** 2D pseudo-color plot of normalized PL spectra as a function of pump fluence. As a help to the reader, PL spectra obtained at the fluence of 50 μJ cm^−2^ and 200 μJ cm^−2^ are also shown (gray curves). **c** Fluen**c**e-dependent emission intensity (red circles), linewidth (blue triangles), and peak energy shift (green squares). The peak energy shift is defined to be a deviation from 2.280 eV. **d**–**f** Color maps of angle-dependent PL spectra obtained under **d** cw-160 mW cm^−2^ excitation, **e** pulsed-17 μJ cm^−2^ pumping (below threshold), and **f** pulsed-100 μJ cm^−2^ pumping (above threshold). The excitation densities expected for these conditions are **d** ~2.9 × 10^15 ^cm^−3^, **e** ~2.7 × 10^18 ^cm^−3^, and **f** ~1.6 × 10^19 ^cm^−3^, respectively. Dotted curves and a dashed line exhibit uncoupled cavity-photon modes and exciton mode, respectively. A thick dashed curve shows a lower branch of coupled modes. **g** Microscopic emission image observed under the above-threshold pumping fluence. Spatial resolution is ~200 nm. A stabilized Michelson interferometer equipped with a retroreflector is employed to observe interference fringes due to long-range phase coherence. The scale bars are 2.5 µm. **h** Occupancy of excited particle deduced from far-field emission data shown in (**b**). Below- (blue square), around- (black circle), and above- (red triangle) threshold data are shown. The horizontal axis shows energy shift from the energy at *k* = 0. Black dashed line exhibits Maxwell–Boltzmann distribution function with *k*_B_*T* = 26 meV. Red dashed curve reveals Bose distribution function with *k*_B_*T* = 26 meV

Next, we show PL results for a CsPbBr_3_ microcavity. Figure 4a exhibits PL spectra obtained under various excitation conditions, which are recorded by measuring the emission integrated within the azimuth of < ~20°. This microcavity has a stop band (R ~99%) of 2.13–2.64 eV (see also Fig. S4a in Supplementary Information). Figure 4b and c summarizes the *I*_p_-dependence of PL signal under the pulse pumping. When we excite the microcavity using a continuous-wave (cw) laser (excitation power of 160 mW cm^−2^, see the dashed curve in Fig. 4a), the observed emission signal is very similar to that of the microplate (see Fig. S5 in Supplementary Information). To characterize this signal, a colormap of angular-resolved PL spectra representing the mode dispersion in in-plane reciprocal space is shown in Fig. 4d. We find four or five dispersion modes around an energy range of the angular-integrated PL signal (2.32–2.42 eV). The angular dispersions of these modes are well described as uncoupled cavity-photon modes represented as $E_{{{{\mathrm{cav}}}}}\left( \theta \right) = E_{{{{\mathrm{cav}}}}}\left( 0 \right)\left( {1 - \sin ^2\theta /n_{{{{\mathrm{eff}}}}}^2} \right)^{ - 1/2}$^20^, where we take into account the wavelength dispersion of background refractive index to explain effective refractive index (*n*_eff_. ~1.5–2.0, see Supplementary Note 3 in Supplementary Information). This fact reveals that the microcavity system under the weak cw excitation is still in a weak coupling regime. This is consistent with a prediction based on the Saha relationship; at the very low excitation density of < ~2.9 × 10^15 ^cm^−3^ (at 160 mW cm^−2^, see Note 2 in Supplementary Information), most of the excited particles in CsPbBr_3_ microcavity are uncorrelated e–h pairs, i.e., free carriers. Due to the small oscillator strength of uncorrelated e–h pairs, the light–matter coupling parameter remains small.

Under the pulse pumping with *I*_p_ of ~17 μJ cm^−2^ (see open circles in Fig. 4a), corresponding to the excitation density of ~2.7 × 10^18 ^cm^−3^, the angular-integrated PL band shows a red shift to a range of ~2.25–2.36 eV. As shown in Fig. 4e, furthermore, the mode dispersion profile exhibits a drastic change from the cw-excitation condition; the uncoupled cavity-photon modes tend to disappear, and another mode exhibiting higher *n*_eff_ emerges instead. The angular dispersion of the newly observed mode, $E_{{{{\mathrm{LB}}}}}\left( \theta \right)$, is well explained by a coupled oscillator model, in which the energy of resonant absorption peak shown in Fig. 2c (*E*_osc_~2.38 eV) is employed as the oscillator coupled with the cavity-photon modes. This dipole coupling causes a formation of lower branch (LB) of the coupled states^19,20^. The Rabi splitting energy of ~270 meV is enough larger than the linewidths of *E*_osc_ and *E*_cav_ (those are estimated to be ~50 meV for both in the absorption peak and the below-threshold emission peak, respectively). The detailed analysis of the coupled oscillator model is described in Supplementary Note 4 and Fig. S6 in Supplementary Information. Based on the analogy of exciton polariton, we can say that the microcavity system is in the strong coupling regime^19,20^. The formation of strong coupling evidently reveals the enhancement of oscillator strength for the excited particles. This idea is consistent with the Saha relationship; at the excitation density larger than ~10^17 ^cm^−3^, which corresponds to *I*_p_ of ~0.6 μJ cm^−2^, a ratio of the strongly correlated e–h pairs (i.e., excitons), having large oscillator strength increases rapidly (see Fig. 1a). The weak-to-strong transition in coupling regimes is first demonstrated in this work, showing a unique feature of the excited state in the CsPbBr_3_ microcavity system.

Above *I*_p_ of ~70 μJ cm^−2^, as shown in Fig. 4b, the PL signal drastically changes from the strong coupling mode to a spectrally narrow emission appearing at ~2.28 eV. Figure 4c reveals the emission intensity showing a S-shaped nonlinear dependence on *I*_p_, with a threshold of ~60 μJ cm^−2^ (see red circles). At the same time, the spectral linewidth is drastically narrowed from ~32 meV to a few meV (see blue triangles). The above-threshold emission exhibits a blue shift in its peak energy (see green squares) and a slight increase in linewidth. We have confirmed the reproducibility of this laser-like behavior as shown in Fig. S4a in Supplementary Information. The energy of laser-like emissions depends on the thickness of CsPbBr_3_ microplate, but is limited to the range of the coupled mode (2.28–2.32 eV). The clear polarization dependence is also observed (Fig. S4b in Supplementary Information). Furthermore, the angular-dependent PL measured at *I*_p_ of ~100 μJ cm^−2^ (~1.6 × 10^19 ^cm^−3^, see Fig. 4f) shows that the PL signal eventually condenses into the energy minimum of the LB curve. Figure 4g shows a microscopic emission image recorded at the above-threshold pumping fluence utilizing a stabilized Michelson interferometer equipped with a retroreflector. Interference fringes generated between the emitted light from the microcavity and its inverted image are clearly observed, whereas no pattern is observed in images at the below-threshold pumping fluence and without the Michelson interferometer setup (Fig. S7 in Supplementary Information), indicating the formation of long-range phase coherence among the excited particles. An interference pattern is observed across the entire emission region, revealing the formation of long-range coherence across a distance of ~4 µm at least. Figure 4h shows the occupancy of the excited particles as a function of energy shift from the energy at *k* = 0. Around the threshold density (*P*_th_ ~60 μJ cm^−2^), the data can be roughly fitted by Maxwell–Boltzmann function at room temperature. Above *P*_th_, the result clearly shows Bose–Einstein statics. Below *P*_th_, on the other hand, the spectrum reveals the distribution of carriers in a wide energy range.

We have confirmed the reproducibility of our central finding for CsPbBr_3_ microcavities with different thicknesses, as shown in Figs. S8 and S9 in Supplementary Information. While there are variations in the threshold fluence and the dispersion characteristics, all samples show very similar features as in Fig. 4d–f, those are the weak-to-strong transition in coupling regime and the condensation into energy minima at the strong coupling regime.

The experimental results shown above evidently reveal a bosonic condensate of photoexcited particles. This scenario is very similar to the work of e.g., Xiong et al.^14,15^, in which they claim polaritonic lasing at room temperature^49–51^. However, it is apparent from the PL results of the bare microplate that the threshold excitation density for the lasing exceeds the Mott density. Therefore, we should identify the observed emission from a condensed polariton BCS state, not a polariton BEC. To confirm this speculation, we discuss the mean separation among the excited e–h pairs, that has been frequently used in recent studies on the polariton BCS for conventional inorganic semiconductor systems^35,36,40^. A dimensionless parameter *r*_s_ (a mean distance between the excited species normalized by the exciton Bohr radius *a*_B_, namely $r_{{{\mathrm{s}}}} = \left( {3/4\pi n} \right)^{1/3}a_{{{\mathrm{B}}}}^{ - 1}$), is a good indicator to distinguish between the BEC and BCS regimes. Shimano et al. have shown in transient absorption experiments that when *r*_s_ is below ~1.4, the dressed photon state due to the light–matter coupling is no longer displayed as a simple excitonic analog to the BEC dressed state and should be assigned as many-body state in the BCS regime^36^. For CsPbBr_3_, as *a*_B_ has been estimated to be ~3.5 nm^52^, *r*_s_ at the excitation conditions of Fig. 4d (cw excitation of 160 mW cm^−2^, *n* = 2.9 × 10^15 ^cm^−3^) is evaluated to be ~12.4. This is large enough to exclude the BCS regime. On the other hand, when the excitation density is increased to *n* = 2.7 × 10^18 ^cm^−3^ and 1.6 × 10^19 ^cm^−3^ (correspond to the pumping conditions of Fig. 4e, f), *r*_*s*_ is reduced to 1.27 and 0.70, respectively. These facts clearly show that strong coupling and energy condensation occur at the BCS regime.

It should be noted that, in the strong coupling regime including the above-threshold fluence region, the “polaritonic” BCS and “excitonic” BCS states may coexist. The polaritonic BCS state is attributed to long-range correlated e–h pair coupled with cavity-photon mode and plays the role of “emitter state” in the microcavity system. On the other hand, the excitonic BCS is the uncoupled e–h pair behaving as “reservoir state” in the system. It is considered that the Mott transition criteria are not the same between these two states, and thus the detailed description of the actual excited state is more complicated. Nevertheless, given that the strong light field in the microcavity is possible to enhance the e–h correlation^35,36^, it is reasonable that the strong coupling remains stable even under a density higher than the Mott density estimated from the result of cavityless bare crystal. Even in such a case, uncorrelated e–h plasma will be possible to play as the reservoir state.

The further increase in the pumping fluence causes the complete Coulomb screening and leads to the e–h plasma regime. In this regime, the microcavity system moves to the weak coupling regime again due to the dissociation of e–h pairs. A normal photon lasing signal is seen as a bundle of PL emissions at ~2.29–2.30 eV (see open arrow in Fig. 4b). Note that we did not measure the angular-dependent PL in this strong pumping regime to avoid the sample degradation under the strong pumping. It would be more helpful to understand the excited states at the high-density region.

It is noteworthy that in CsPbBr_3_ microcavity, the polariton BCS was successfully observed at RT. This is in contrast to recent works on GaAs-based microcavities where the polariton BCS is investigated at cryogenic temperature^36,39,40^. As a future work, it is expected to find a more solid evidence of the RT polariton BCS, e.g., observation of BCS-like energy gap.

## Discussion

We investigated the excited states of CsPbBr_3_ microcavity system at the below- and above-threshold pumping conditions. An important finding in this work is a transition in the coupling regime, which is observed as a drastic change in the PL dispersion curve depending on the pumping fluence. This exhibits a change in the dominant excited species from uncorrelated e–h pair to exciton. At the pumping fluence above the threshold, PL signal from the CsPbBr_3_ microcavity system shows lasing behaviours with bosonic energy condensation characteristics. On the other hand, PL signal from a cavityless CsPbBr_3_ microplate under this fluence range exhibits features of emission from uncorrelated e–h plasmas. These results demonstrate that the condensed polariton BCS is as origin of the strong coupling formation and the lasing behavior. In this work, the RT polariton BCS and its energy condensation is first demonstrated in lead-halide perovskite material. This fact will open new insights into the quantum device applications of the light–matter strong coupling systems.

## Materials and methods

### Preparation of precursor solutions

CsBr and PbBr_2_ were purchased from Tokyo Chemical Industry and used as received. They were dissolved in DMSO at a concentration of 0.4 M. The solution was stirred at 300 rpm and 50 °C for 10 min. After that, ACN was added as a poor solvent until the solution reached saturation. The solution was stirred at room temperature for 3 h and subsequently filtered.

### Crystal growth

CsPbBr_3_ microplates were grown by the antisolvent vapor-assisted crystallization method^41^. Two silica plates (area: 10 × 10 mm^2^ and thickness 0.5 mm) were cleaned with acetone, ethanol, and isopropyl alcohol in an ultrasonic bath for 5 min for each solvent. One of the plates was exposed to UV ozone to obtain a hydrophilic surface. The other was spin-coated with hexamethyldisilazane in a two-step sequential condition (500 rpm for 5 s followed by 3000 rpm for 10 s) to obtain a hydrophobic surface. In total, 10 µl of the perovskite precursor solution mentioned above was cast on the hydrophilic substrate. The hydrophobic substrate was put on the solution, and then the two plates were pinched with a bulldog clip. The sample was placed in a glass beaker (100 ml), into which 3 ml of ACN was dropped. The glass beaker was sealed with cling film and was put on a hotplate (40 °C) to fill the inner space of the beaker with the ACN mist. The ACN mist penetrated the gap between the two plates slowly and promoted the nucleation of CsPbBr_3_, resulting in microplates after one day. We could control the thickness of the microplate by limiting the vertical space with a pair of the plates (i.e., space-limited antisolvent vapor-assisted crystallization method)^31^. The lateral size and thicknesses of crystals were evaluated by a scanning electron microscope (TM3030 plus, Hitachi) and a surface profiler (Dektak XT-S, Bruker) observations, respectively, after removing the top plate. Typically, the microplates showed square or rectangular shapes with a dimension of 50–200 μm, and their thickness is in a range of 200 nm–3.5 μm. X-ray diffraction measurements (D8 Discover, Bruker) showed two distinct peaks at 15.2° and 30.5°, which are attributed to diffractions of (100) and (200) planes, respectively, and consistent with a recent report on the cubic or orthorhombic CsPbBr_3_ microplate^43^.

### Microcavity fabrication

To fabricate microcavities, glass plates on which DBRs were deposited were used instead of the untreated silica plates. The DBRs were multilayers of SiO_2_ and TiO_2_ on BK7 glass plates (area: 10 × 10 mm^2^ and thickness 0.5 mm). The rf-magnetron sputtering method was employed for the deposition of nine pairs of SiO_2_ and TiO_2_ layers. The resultant DBR had a reflectivity larger than 99%. In this study, we used two types of DBRs (see Fig. S4a in the Supplementary Information). One of them had a reflection band of ~450–550 nm and was used as the top mirror. The other had a reflection band of ~500–600 nm and was used as the bottom mirror. This combination enabled the formation of microcavity with a band of 2.1–2.6 eV as well as good transparency for excitation light (405 nm or 351 nm).

### Optical characterizations

All optical measurements were performed in air at room temperature (~23 °C) and relative humidity of ~40%RH. For the steady-state photoluminescence measurements, a cw-laser diode of 405 nm was used for the sample excitation. The excitation density was ∼30 mW cm^−2^. The emission was collected using a quartz optical fiber with a core diameter of 1 mm. The emission spectra were measured with a CCD spectrometer with a resolution as high as ∼0.3 nm (Triax 550 & Synapse, Horiba). The emission counts were measured with time-integration for 1 s. For pulse pumped PL measurements, the third harmonic generation of the Nd: YLF ~8-ns pulse laser with a wavelength of 351 nm and a repetition rate of < 10 Hz (Quantas-Q1D-TH-ATF, Quantum Light Instruments) was used as an excitation source. In the angle-resolved PL measurements, the laser light was focused using a plano-convex lens with a focal length of 200 mm. The detection angle of emission was varied by using a homemade rotational stage on which an optical fiber (core diameter of 1 mm) for collecting the emission was placed.

## Supplementary information

Supplementary Information

## Data Availability

The datasets generated during and/or analyzed during the current study are available from the corresponding author on reasonable request.
