# Supplementary material for: Drastic transitions of excited state and coupling regime in all-inorganic perovskite microcavities characterized by exciton/plasmon hybrid natures
Source: Light Sci Appl. 2022 Jan 2;11:8. doi: 10.1038/s41377-021-00701-8 (PMC8720309; doi:10.1038/s41377-021-00701-8)
Supplement: Supplementary file 1 — Supplementary Information [file 41377_2021_701_MOESM1_ESM.docx]

Supplementary Information for

Drastic transitions of excited state and coupling regime in all-inorganic perovskite microcavities characterized by exciton/plasmon hybrid natures

S. Enomoto, T. Tagami, Y. Ueda, Y. Moriyama, K. Fujiwara, S. Takahashi, and K. Yamashita

**Contents**

**1. Excitation density and relevant physics**

*1-1. Calculation of exciton density in CsPbBr_3_ based on Saha relationship*

*1-2. Estimation of excitation density under pulse pumping and cw excitation*

**2. Structural characterization of CsPbBr_3_ microplates**

**3. Optical characterizations of CsPbBr_3_ microplates**

*3-1. Random lasing at high pumping fluence*

*3-2. PL characterization of microcavities*

**4. Optical characterizations of CsPbBr_3_ microcavities**

*4-1. Angular dispersion of uncoupled cavity modes in CsPbBr_3_ microcavity*

*4-2. Coupled oscillator model describing polariton dispersion*

*4-3. Evaluation of spatially long-range phase coherence*

*4-4. Reproducibility check of PL results for full-VCSEL*

**1. Excitation density and relevant physics**

*1-1. Calculation of exciton density in CsPbBr_3_ based on Saha relationship*

**Note 1** The Saha relationship that describes the thermal equilibrium of correlated and uncorrelated e-h pairs excited in semiconductor materials is shown as follows.

| $x=\left( n+\frac{A}{2} \right)-\sqrt{\left( \frac{A}{2} \right)^{2}+An}$ | (S1.1) |
| --- | --- |

Here $x$ and $n$ are exciton density and the total density of excited species, and

| $A=\left( \frac{\sqrt{2\pi m_{e}k_{B}T}}{h} \right)^{3}\exp\left( -\frac{E_{b}}{k_{B}T} \right)$ | (S1.2) |
| --- | --- |

where $k_{B}$ and $h$ are the Boltzmann constant and Planck’s constant, respectively, and $m_{e}$ is electron mass. Figure 1a in the main manuscript shows calculated results of exciton fraction ($x/n$) at room temperature (RT, $T$ = 300 K). For CsPbBr_3_ showing the exciton binding energy of ~30 – 60 meV at RT, $x/n$ increases rapidly above $n$ of ~10^16^ cm^−3^ and reaches 0.5 at $n$ of ~2 – 5 × 10^17^ cm^−3^. This excitation density corresponds to the pump fluence around ~1.3 – 3.1 µJ cm^−2^.

*1-2. Estimation of excitation density under pulse pumping and cw excitation*

**Note 2** The excitation density $n_{\mathrm{pulse}}$ under pulse pumping with the fluence of $I_{p}$ at the energy of $E_{\mathrm{ph}}$ is simply estimated from the following relationship.

| $n_{\mathrm{pulse}}=\frac{\alpha I_{p}}{E_{\mathrm{ph}}d}$ | (S1.3) |
| --- | --- |

Here $d$ is the thickness of sample measured (~100 nm) and $\alpha$ (~0.88) is absorption ratio of the pumping light. $\alpha$ was estimated from incident, reflected, and transmitted light powers. We use a 351-nm light source. From (S1.3), $n$ is estimated to be ~1.6 × 10^18^ cm^−3^ at $I_{p}$ of ~10 μJ cm^−2^.

On the other hand, the excitation density $n_{\mathrm{cw}}$ under cw excitation with the power density of $P_{\mathrm{cw}}$ is estimated from the following relationship.

| $n_{\mathrm{cw}}=\frac{\alpha P_{\mathrm{cw}}\tau}{E_{\mathrm{ph}}d}$ | (S1.4) |
| --- | --- |

Here $\tau$ is lifetime of the excited species. As shown in Fig. S1, $\tau$ of CsPbBr_3_ microcavity is evaluated from time-resolved PL measurement to be ~100 ns. Thus, from (S1.4), $n_{\mathrm{cw}}$ under cw excitation is estimated to be ~2.9 × 10^15^ cm^−3^.

To perform the time-resolved PL measurements, the emission was dispersed in a monochromator (SPG-120SS, SHIMADU) and detected by a single-photon detector (ID-100, ID Quantique). The signal was collected with a time-correlated single photon counting module (SPC-130EM, Becker & Hickl GmbH).

**

**Figure S1**. Time-resolved PL result for CsPbBr_3_ microcavity. The excitation light source is 405-nm laser diode operated at 1 MHz. Solid line shows a single-exponential function.

**2. Structural characterization of CsPbBr_3_ microplates**

We obtain CsPbBr_3_ microplates by antisolvent vapor-assisted crystallization method. Figure S2a shows a scanning electron microscopic (SEM) image of one of the microplates, which has a square-shaped crystal slab having a flat surface. We evaluated the dimensions of microplate with surface profiler measurements as shown in Fig. S2b. Typically, the microplates have lateral dimension of 50 – 200 μm, and thickness in a range of 200 nm – 3.5 μm. X-ray diffraction spectrum shown in Fig. S2c exhibits two distinct characteristic peaks at 15.2 º and 30.5 º assigned to (110) and (220) planes, respectively, of orthorhombic phase. The relatively small intensities of other diffraction peaks reveal the [110]-oriented growth. However, as peak splitting [(110)/(002) and (220)/(004) splitting] are not clearly seen,^S1^ a possibility of cubic phase growth cannot be excluded.

**Figure S2**. **a** Scanning electron microscopic image of CsPbBr_3_ microplates. Dashed line indicates a trace line for a surface profiler measurement. **b** Some typical results of surface profiling of CsPbBr_3_ microplates. The profile indicated by “A” and “B” is of the microplate shown in **a**. **c** X-ray diffraction spectrum of a CsPbBr_3_ microplate.

**3. Optical characterizations of CsPbBr_3_ microplates**

*3-1. Random lasing at high pumping fluence*

PL properties of CsPbB_3_ microplate under high pumping fluence (> ~100 μJ cm^−2^) have been investigated. As shown in Fig. S3a, the PL spectrum exhibits a variation in its profile; the relative intensity of a low-energy PL component (blue curves) tends to increase with the pumping fluence. This behaviour is clearly found also in Fig. 2b in the main manuscript. When the pumping fluence exceeds ~100 μJ cm^−2^, new emission modes appear around ~2.28 eV (see the top spectrum in Fig. S3a). We find in a high-resolution measurement (resolution of ~0.1 nm, see Fig. S3b) that these emission modes consist of a lot of fine structure having a mode separation of ~1 nm. Given that the free spectral rage is ~28 cm^-1^, random lasing is the most possible origin for the observed emission.

**Figure S3**. **a** Pumping fluence dependence of PL spectrum for CsPbBr_3_ microplate. Spectra are decomposed into two PL signals (blue and green curves). **b** Result at high-resolution measurement.

*3-2. PL characterization of microcavities*

Reflectivity spectra of DBRs used in this study are shown in Fig. S4a. The top DBR has a 99-% reflection band of ~450 – 550 nm whereas the bottom DBR has that of 500 – 600 nm. The emission wavelength of CsPbBr_3_ microplate is included in the reflection band of microcavity (2.1 – 2.6 eV). At the above threshold condition, microcavity samples show laser-like emission. The peak energy of lasing depends on the crystal thickness. We have confirmed the lasing emission strongly polarized to one of the in-plane directions of the VCSEL microcavity as shown in Fig. S4b. We find that the polarization extinction ratio is larger than 10:1, demonstrating the high coherence of condensed polariton state.

**Figure S4**. Above-threshold PL characterization of full-VCSEL samples with CsPbBr_3_ microplates. **a** Lasing spectra at above-threshold pumping are shown by red, green, blue, and purple curves for microcavity samples with the crystal thicknesses of 170, 630, 700, and 810 nm, respectively. For comparison, PL spectrum of a microplate sample is exhibited by a dashed curve. Pink and light purple curves show reflectivity spectra of top and bottom DBRs respectively. **b** Polarization dependence of the lasing emission spectrum. Inset shows a polar plot of integrated intensity as a function of polarization angle.

*3-3. cw-PL characterizations*

Figure S5 shows cw-excited PL spectra of CsPbBr_3_ microplate and microcavity. The excitation wavelength and density were 405 nm and 160 mW cm^−2^, respectively. The spectral profiles are very similar in the samples with and without cavities. This result is due to the fact that the microcavity system is in the weakly coupled regime, in which multiple cavity photon modes showing angle-sensitive dispersion curves can contribute to the emission and the excited species are the same as those in the microplate (uncorrelated free carriers). Under such a case, with or without cavity, the energy-distributed excitation can be out-coupled to electromagnetic modes with various energies (vacuum fields for the bare microplate and cavity photon modes for the microcavity), so that the emission spectra directly reflect the energy profile of the excited carriers. In other words, we can confirm that the microcavity under the cw excitation is definitely in the weak coupling regime.

**Figure S5**. cw-excited PL spectra of CsPbBr_3_ microplate (blue curve) and microcavity (red curve). The excitation wavelength and density were 405 nm and 160 mW cm^−2^, respectively.

**4. Optical characterizations of CsPbBr_3_ microcavities**

*4-1. Angular dispersion of uncoupled cavity modes in CsPbBr_3_ microcavity*

**Note 3** In a microcavity, as the vertical component of wavevector for photon is quantized, the energy of cavity photon mode is discretized and their dependence on the observation angle $\theta$ is expressed as follows.

| $E_{\mathrm{ph}}\left( \theta\right)=\frac{m\pi\hbar c}{n_{\mathrm{eff}}L}\left( 1-\frac{\sin^{2} \theta}{n_{\mathrm{eff}}^{2}} \right)^{-\frac{1}{2}}$ | (S4.1) |
| --- | --- |

Here $L$ is the cavity length, $m$ is the mode number, and $c$ is the light velocity in vacuum. An important parameter to analyse the experimental observation is the effective refractive index $n_{\mathrm{eff}}$. In a microcavity, $n_{\mathrm{eff}}$ is approximately determined by the overlap integral between the electric field distribution function and the material index profile consisting of the perovskite active layer and dielectrics in DBRs (SiO_2_ and TiO_2_ having material indices of 1.46 and 2.49, respectively). Furthermore, as the electric field of cavity photon mode spreads into the DBRs deeply, we need to consider also the contribution of DBRs. In addition, the material index of perovskite also shows a strong dispersion, especially in the wavelength range near the band gap (*n* ~ 2 – 2.4, see Ref. S2). In the current case, we find that the five cavity modes observed in Fig. 4c of the main manuscript can be reasonably explained by taking $n_{\mathrm{eff}}$ = 1.49, 1.54, 1.62, 1.71, and 2.00 where $m$ = 9, 10, 11, 12, and 13, respectively.

*4-2. Coupled oscillator model describing polariton dispersion*

**Note 4** Coupling between the cavity photon mode and exciton transition dipole moment is often described by the coupled oscillator model using a phenomenological Hamiltonian $H$ shown as follows.

| $H=\left[ \begin{matrix} E_{\mathrm{cav}}\left( \theta\right) & \frac{\hbar\Omega}{2} \\ \frac{\hbar\Omega}{2} & E_{\mathrm{osc}} \end{matrix} \right]$ | (S4.2) |
| --- | --- |

Here $E_{\mathrm{osc}}$ is the energy of transition dipole moment and is treated as constant (~2.38 eV) because their dispersion curve is much less sensitive to $\theta$ than $E_{\mathrm{ph}}$. $\hbar\Omega$ is Rabi-splitting energy that indicates strength of the coupling, and can be represented as follows.

| $\hbar\Omega=\sqrt{4V^{2}-\left( \gamma_{\mathrm{cav}}-\gamma_{\mathrm{osc}} \right)^{2}}$ | (S4.3) |
| --- | --- |

Here $V$ is coupling parameter. Damping constants, $\gamma_{\mathrm{cav}}$ and $\gamma_{\mathrm{osc}}$, correspond to the imaginary parts of $E_{\mathrm{cav}}$ and $E_{\mathrm{osc}}$, respectively,^S3^ and agree with the energetic linewidth of these modes (~50 meV). The energies of polariton modes are obtained by solving an eigenvalue problem for $H$. As the result the lower polariton energy $E_{\mathrm{pol}}^{-}$ are written as follows.

| $E_{\mathrm{pol}}^{-}=\frac{\left( E_{\mathrm{cav}}+E_{\mathrm{osc}} \right)-\sqrt{\left( E_{\mathrm{cav}}-E_{\mathrm{osc}} \right)^{2}+\left( \hbar\Omega\right)^{2}}}{2}$ | (S4.4) |
| --- | --- |

Note that $E_{\mathrm{cav}}$ depends on $\theta$ as shown by Eq. (S4.1). By using Eq. (S4.4), we have performed a fitting analysis for the experimentally obtained $E_{\mathrm{pol}}^{-}$, as shown in Fig. S6. The coupling between one of the $E_{\mathrm{cav}}$ modes, having $n_{\mathrm{eff}}$ = 2.00 and $m$ = 13, and the $E_{\mathrm{osc}}$ mode can well explain the experimental data. As a result, $\hbar\Omega$ is evaluated to be ~270 meV.

**Figure S6**. Fitting analysis of angular dependent polariton emission by using a coupled oscillator model. Red open circle shows PL peak energy those are extracted from the results of Fig. 4e in the main manuscript. Dashed line and dotted curve exhibit the transition dipole moment energy ($E_{\mathrm{osc}}$ ~ 2.38 eV) and uncoupled cavity photon mode ($E_{\mathrm{cav}}$) with $n_{\mathrm{eff}}$ = 2.00 and $m$ = 13, respectively. Dashed red curve shows a fitting result using a function of Eq. (S4.4) ($E_{\mathrm{pol}}^{-}$). In this analysis, $\hbar\Omega$ is treated as the only fitting parameter and is estimated to be ~270 meV.

*4-3. Evaluation of spatially long-range phase coherence*

We measured long-range coherence of above-threshold emission from the strongly-coupled state in CsPbBr_3_ microcavity. This evaluation was performed by using a Michelson interferometer setup where one of the interferometer arms is equipped with a retroreflector. Figure S7 shows colour maps of real-space images of photoexcited area. At pumping fluence below the threshold, the photoexcited area simply shows a diffused emission pattern in both cases for single-arm (Fig. S7a) and double-arm (Fig. S7b) configurations. Above threshold, on the other hand, interference fringes are clearly observed only at the double-arm configuration (Fig. S7d) whereas the single-arm configuration shows a simple Gaussian-like profile (Fig. S7c). These results evidently show the long-range phase coherence of the condensed particles.

**Figure S7**. Evaluation of long-range phase coherence using a Michelson interferometer setup. **a** and **b** Colour maps of emission profiles at below-threshold pumping fluence. **c** and **d** Colour maps of emission profiles at above-threshold pumping fluence. **a** and **c** The emission profiles recorded at single-arm configuration. **b** and **d** The emission profiles recorded at double-arm configuration.

*4-4. Reproducibility check of PL results for full-VCSEL*

We have examined reproducibility check for the results shown in Figs. 4c – 4f of the main manuscript. The PL spectra depending on the pumping fluence and the observation angle have been measured for the other two VCSEL samples with crystal thicknesses of ~200 nm and ~1.4 µm, as summarized in Figs. S8 and S9, respectively. In the former sample, spectral narrowing appears at $I_{p}$ ~12 µJ cm^−2^ (Fig. S8a). At the same time, PL intensity shows a sudden increase (Fig. S8b). In the angular dependent measurement, the PL signals showing the dispersion of uncoupled cavity mode has not been clearly observed (Fig. S8c), probably due to the small crystal thickness and the large spectral mode separation. With increased the pumping fluence, however, energy condensation and the following blue shift are clearly observable (Figs. S8d and S8e). The other sample having the crystal thickness of ~1.4 µm shows similar results. At the low pumping fluence, the transition point from the weak coupling to the strong coupling is clearly observed (Fig. S9c). The threshold for the spectral narrowing and drastic PL increase is ~100 µJ cm^−2^ (Fig. S9b). It should be noted that the sample with a thinner crystal thickness exhibits a lower threshold, implying that the efficient energy transfer from the reservoir to the polariton branch is obtained by limiting the number of modes. This might be reasonable because the radiative energy transfer between the reservoir and polariton branch would depend on the density of state of polariton branch.

**Figure S8**. Polariton lasing in a microcavity of CsPbBr_3_ microplate with a thickness of ~200 nm. **a** Comparison of PL spectra obtained at three different pumping fluence. **b** Fluence-dependent PL intensity (pink circles), emission line width (blue triangles), and peak position (green squares). **c** – **e** PL contour maps of angular dependent PL spectra at different pumping conditions.

**Figure S9**. Polariton lasing in a microcavity of CsPbBr_3_ microplate with a thickness of ~1.4 µm. **a** Comparison of PL spectra obtained at three different pumping fluence. **b** Fluence-dependent of PL intensity (pink circles), emission line width (blue triangles), and peak position (green squares). **c** – **e** PL contour maps of angular dependent PL spectra at different pumping conditions.

References in Supplementary Material

1. Zhang, M., Zheng, Z., Fu, Q., Chen, Z., He, J. Zhang, S., Yan, L., Hua, Y., and Luo, W. Growth and characterization of all-inorganic lead halide perovskite semiconductor CsPbBr_3_ single crystals. *CrystEngComm* **19**, 6797-6803 (2017).
2. Zhao, M., Shi, Y., Dai, J., and Lian, J. Ellipsometric study of the complex optical constants of a CsPbBr3 perovskite thin film. J. Mater. Chem. C 6, 10450-10455 (2018).
3. Nishimura, S., Yamashita, K., Takahashi, S., Yamao, T., Hotta, S., Yanagi, H., and Nakayama, M. Quantitative evaluation of light–matter interaction parameters in organic single-crystal microcavities. *Opt. Lett.* **43**, 1047-1050.
